# Menopause-associated risk of cardiovascular disease

**DOI:** 10.1530/EC-21-0537

**Published:** 2022-03-08

**Authors:** Panagiotis Anagnostis, Irene Lambrinoudaki, John C Stevenson, Dimitrios G Goulis

**Affiliations:** 1 Unit of Reproductive Endocrinology, 1st Department of Obstetrics and Gynecology, Medical School, Aristotle University of Thessaloniki, Thessaloniki, Greece; 2 2nd Department of Obstetrics and Gynecology, National and Kapodistrian University of Athens, Medical School, Athens, Greece; 3 National Heart and Lung Institute, Imperial College London, Royal Brompton and Harefield Hospitals, Guy’s and St Thomas’ NHS Foundation Trust, London, UK

**Keywords:** menopausal hormone therapy, early menopause, premature ovarian insufficiency, cardiovascular disease

## Abstract

Cardiovascular disease (CVD) is of major concern in women entering menopause. The changing hormonal milieu predisposes them to increased CVD risk, due to a constellation of risk factors, such as visceral obesity, atherogenic dyslipidemia, dysregulation in glucose homeostasis, non-alcoholic fatty liver disease and arterial hypertension. However, an independent association of menopause *per se* with increased risk of CVD events has only been proven for early menopause (<45 years). Menopausal hormone therapy (MHT) ameliorates most of the CVD risk factors mentioned above. Transdermal estrogens are the preferable regimen, since they do not increase triglyceride concentrations and they are not associated with increased risk of venous thromboembolic events (VTE). Although administration of MHT should be considered on an individual basis, MHT may reduce CVD morbidity and mortality, if commenced during the early postmenopausal period (<60 years or within ten years since the last menstrual period). In women with premature ovarian insufficiency (POI), MHT should be administered at least until the average age of menopause (50–52 years). MHT is contraindicated in women with a history of VTE and is not currently recommended for the sole purpose of CVD prevention. The risk of breast cancer associated with MHT is generally low and is mainly conferred by the progestogen. Micronized progesterone and dydrogesterone are associated with lower risk compared to other progestogens.

## Introduction

Cardiovascular disease (CVD) is the leading cause of death in women, involving 50% of cases, with 20% attributed to ischemic heart disease (IHD) and 13% to stroke (1). This is also the case for women younger than 65 years, with 26% of deaths assigned to CVD (1). Except for obstructive coronary heart disease (CHD), other major causes of IHD in middle-aged women include coronary artery spasm and coronary microvascular dysfunction (2). Although IHD in females occurs 7–10 years later compared with males, mostly due to the protective effect of estrogens on the atherosclerotic process, there is a steady increase in this risk after the transition to menopause (2). This is mostly evident in women with early menopause (EM; defined as age at menopause <45 years) or premature ovarian insufficiency (POI; defined as age at menopause <40 years) (3).

The aim of this narrative review was to provide an evidence-based approach to the menopause-associated CVD risk. Moreover, the effect of menopausal hormone therapy (MHT) on this risk is also discussed.

## Does transition to menopause predispose to higher CVD risk?

Menopause, defined as the completion of 12 months since the final menstrual period (FMP) or at the time of bilateral oophorectomy, is the consequence of follicle depletion which results in estrogen deficit (4). Epidemiological evidence has shown that menopausal transition is associated with a higher prevalence of CVD risk factors, such as central adiposity, atherogenic dyslipidemia, glucose intolerance, arterial hypertension (AH) and non-alcoholic fatty liver disease (NAFLD), compared with premenopausal status (5).

In detail, transition to menopause leads to body fat redistribution toward the male pattern of visceral adiposity (6, 7). Indeed, the onset of menopause is followed by a reduction in fat oxidation and a decrease in energy expenditure, without changes in energy intake (7). In studies using dual-energy X-ray absorptiometry, CT or MRI, postmenopausal women have 36% more thoracic fat and 49% greater intra-abdominal fat area compared with premenopausal women (8). These differences were independent of age and total fat mass (8). Moreover, biopsy studies in postmenopausal women have shown hypertrophy of adipocyte cells both in subcutaneous and visceral adipose tissue, as well as increased inflammation and fibrosis, compared with premenopausal women (9). One mechanism for the postmenopausal body fat redistribution may be the upregulation of adipose tissue lipoprotein lipase activity and a lower degree of lipolysis, due to the decrease in estrogen concentrations (10, 11). The loss of 17β-estradiol (17β-E_2_) activation of estrogen receptor (ER) type α (ERα) in neurons of the ventromedial nucleus of the hypothalamus, which regulates adipose tissue distribution, constitutes another mechanism (12).

These changes in adipose tissue may lead to increased insulin resistance (up to 50%) in postmenopausal compared with premenopausal women (13). ERα and ER type β (ERβ) promote β-cell survival and secretion, as shown in animal studies (7). Pancreatic insulin secretion is also reduced by 50% in postmenopausal compared with premenopausal women, irrespective of BMI and age (13). Except for the pancreas, estrogen acts on the liver (via ERα) and reduces gluconeogenesis (14). It also increases glucose uptake in fatty tissue and muscles, mainly via translocation of glucose transporter 4 (14).

On a clinical level, plasma glucose concentrations may be either not affected (13) or dysregulated toward impaired glucose tolerance (IGT) in women who enter menopause (15). The estimated annual incidence of IGT after menopause is 6%, independent of BMI, waist-hip ratio, blood pressure, family history of diabetes mellitus (DM) type 2 (T2DM), age at menopause and lipid profile (15). However, a recent meta-analysis showed that postmenopausal women with a history of EM or POI demonstrate a higher risk for T2DM, compared with those with a normal age at menopause (>45 years) (odds ratio (OR) 1.12, 95% CI 1.01–1.20 and 1.53, 95% CI 1.03–2.27), respectively (16).

Regarding blood pressure (BP), epidemiological evidence suggests an increase after the onset of menopause (17). However, it is not clear if this is a consequence of menopause *per se* or the aging process, due to reduced vascular elasticity and increased prevalence of atherosclerosis in older ages (6). The contribution of other factors, such as obesity, smoking and low physical activity should be taken under consideration (17). In any case, a steeper increase in systolic BP in postmenopausal women (18) has been reported as well as a higher sympathetic activity compared with their male counterparts (19). Furthermore, a recent meta-analysis showed a slight but significant increase in the risk of AH in women with EM compared with those with an age at menopause >45 years (OR 1.10, 95% CI 1.01–1.19) (20). Plausible pathogenetic mechanisms include the production of vasoconstrictive factors, such as endothelin and angiotensinogen, as the result of the decline in estrogen concentrations, and a lower estrogen-to-androgen ratio during menopause (21).

What is of importance regarding the menopause-associated CVD risk is the changes in lipid profile during the transition to menopause. Concisely, these include an increase in total cholesterol (TC), low-density lipoprotein cholesterol (LDL-C), triglycerides (TG), and a decrease in high-density lipoprotein cholesterol (HDL-C) concentrations (22). The latter is mainly attributed to the HDL_2_-C subfraction (22). Notably, except for these changes in lipid profile, atherogenic changes in apolipoprotein concentrations and their ratios have also been reported after the onset of menopause. These include an increase in apolipoprotein B (apoB) concentrations and LDL-C/apoB ratio in postmenopausal women, which are evident from the age of 50–55 years, converging with and exceeding the respective values in men (23). Moreover, despite the rise in apolipoprotein A-I (apoA-I) and apolipoprotein A-II (apoA-II) concentrations in postmenopausal compared with premenopausal women, the HDL-C/apoA-I and HDL-C/apoA-II ratios decrease to the lowest degree seen in men, suggesting a lower cholesterol content of HDL particles (23). Regarding lipoprotein (a) (Lp(a)), an independent risk factor for atherosclerotic CVD (24), inconclusive data exist as to whether this increases after menopause (25).

Furthermore, dyslipidemia and insulin resistance facilitate an increased influx of free fatty acids to the liver and enhance the development of NAFLD (26). Except for estrogen deficit, the relative androgen excess and decrease in sex hormone-binding globulin also contribute to increased abdominal adipose mass and ensuing NAFLD (27). Postmenopausal women are at a two-fold increased risk of NAFLD compared with premenopausal women (27). The respective prevalence of NAFLD in women <45 years, 45–55 and >55 years is 5.3, 18.8 and 27.8%, respectively (28). In cases of obesity, it rises to 48.4% (28). More than 50% of postmenopausal women with T2DM suffer from NAFLD (26). The prevalence of non-alcoholic steatohepatitis is also high in women >55 years (13.2%) and even higher in those with obesity and NAFLD (14.9%) (28).

## Is there an independent association between menopause and increased risk of CVD events?

The data mentioned before suggest an acquisition of an atherogenic profile in women during and after the transition to menopause predisposing them to increased CVD risk. Interestingly, endothelial dysfunction starts in the early postmenopausal period, before signs of subclinical atherosclerosis occur, possibly accounting for the ‘undetermined’ chest pain and dyspnea, often attributed to stress or to menopausal symptomatology (2). In addition, these women are at two-fold increased risk of IHD (2). Inflammatory co-morbidities, such as autoimmune rheumatic (i.e. rheumatoid arthritis, systemic lupus erythematosus) and endocrine disorders (thyroid dysfunction) augment CVD risk in women around menopause (2).

Whether this increased risk is translated into an equivalent risk of CVD events in postmenopausal women, irrespective of the effect of chronological aging, has not been established, as the relevant studies show inconsistent data (29, 30, 31). On the other hand, both EM and POI have been associated with increased CVD morbidity and mortality, mainly due to IHD. According to a meta-analysis, published in 2016 (32 studies; *n* = 310,329 postmenopausal women), the history of EM is associated with a 1.5-fold increased risk for IHD (relative risk (RR) for overall IHD 1.50, 95% CI 1.28–1.76) compared with normal age at menopause (>45 years) (32). This was also the case for fatal IHD (RR 1.11, 95% CI 1.03–1.20), CVD mortality (RR 1.19, 95% CI 1.08–1.31) and all-cause mortality (RR 1.12, 95% CI 1.03–1.21). However, no association with overall stroke risk and stroke mortality was observed (32).

With respect to POI, two meta-analyses published in 2016, confirmed these results. In particular, the history of POI augments the risk of all-cause and IHD mortality by 39% (pooled RR 1.39, 95% CI 1.10–1.77) and 48% (pooled RR 1.48, 95% CI 1.02–2.16), respectively, compared with normal age at menopause (>45 years) (33). This was also the case with another meta-analysis (10 studies; *n* =190,588 postmenopausal women), showing an increased risk of IHD (hazard ratio (HR) 1.69, 95% CI 1.29–2.21) and total CVD morbidity or mortality (HR 1.61, 95% CI 1.22–2.12) (34). As with EM, the history of POI was not associated with an increased risk of stroke (33, 34). A recent cohort study from the UK (*n*  = 144,260 postmenopausal women, aged 40–69 years) showed an increased risk for CVD for natural and surgical premature menopause (<40 years) compared with menopause at an age >40 years (HR 1.36, 95% CI 1.19–1.56 and 1.87, 95% CI 1.36–2.58, respectively, after adjustment for conventional CVD risk factors and the use of MHT). This was again mainly attributed to IHD (35).

## Does menopausal hormone therapy reduce CVD risk?

### Menopausal hormone therapy and CVD risk factors

Since menopause augments CVD risk, at least in women with EM and POI, the spontaneously arising question is whether MHT could reduce this risk. Accumulative body of evidence supports the notion that MHT may ameliorate most CVD risk factors, such as visceral adiposity, dyslipidemia and glucose homeostasis to various extent, depending on the formulation used (estrogen type, dose, route of administration and type of progestogen) (6). Briefly, estrogen may decrease TC, LDL-C, Lp(a) and increase HDL-C concentrations in a dose-dependent manner (6, 7, 36). These changes are more pronounced with conjugated equine estrogen (CEE) compared with 17β-E_2_, the latter being higher with oral than with transdermal regimen (6, 7, 36). However, TG concentrations may increase with oral estrogen, whereas they may either decrease or remain stable with the transdermal route (6, 7). Nevertheless, the latter does not affect the coagulation system and is not associated with an increased risk of venous thromboembolism (VTE) in contrast to the oral regimen (37, 38).

MHT may exert either a slight reduction or no effect on BP and BMI (6, 7) and it may reduce visceral adiposity and waist circumference (6, 7). Regarding glucose metabolism, MHT improves glucose homeostasis, by increasing insulin sensitivity and secretion, as well as glucose uptake by the muscles (7). It may also reduce the risk of T2DM by 30% (7, 38). Both oral and transdermal estrogen demonstrate a favorable effect on glucose metabolism, although oral CEE exert a more pronounced effect at equivalent doses (7). Regarding the effect of MHT on NAFLD, current evidence shows inconclusive results (26).

Concerning progestogens, they seem to modify the effect of estrogen on the CVD risk factors mentioned above. Medroxyprogesterone acetate (MPA) and levonorgestrel may attenuate this effect, whereas low-dose norethisterone acetate and dydrogesterone are neutral (7). In general, micronized progesterone or dydrogesterone are the preferred progestogens due to their neutral effect on lipid profile (39).

### MHT and CVD events

Regarding CVD events, several observational studies, especially during the period 1980–2000, have shown a beneficial effect of MHT on CHD risk (40, 41). However, the concept of CVD primary prevention by MHT had to be tested in a randomized controlled trial setting (RCTs). The hallmark RCT, designed to investigate the effect of MHT on CVD (with CHD as the main outcome) and breast cancer risk, was the Women’s Health Initiative (WHI) Study. This study had two arms; the first (WHI-1) compared the effect of CEE 0.625 mg/day plus MPA 2.5 mg/day (*n*  = 8506) with that of placebo (*n*  = 8102) in postmenopausal women (50–79 years old) with an intact uterus (42). This study was early terminated (at 5.2 years) due to evidence of increased risk of invasive breast cancer (HR 1.26, 95% CI 1.00–1.59). The preliminary results from the study showed that the estimated HR for IHD, total CVD, stroke and VTE was 1.29 (95% CI 1.02–1.63), 1.22 (95% CI 1.09–1.36), 1.41 (95% CI 1.07–1.85) and 2.11 (95% CI 1.58–2.82) and, respectively. However, MHT was associated with a reduced risk of colorectal cancer (HR 0.63, 95% CI 0.43–0.92) hip (HR 0.66, 95% CI 0.45–0.98) and total fractures (HR 0.76, 95% CI 0.69–0.85) (42). Notably, when the final results of WHI-1 were published, the risk for CHD risk was not significant (HR 1.24, 95% CI 0.97–1.60) (43).

The second arm (WHI-2) recruited 10,739 postmenopausal women, aged 50–79 years, with a history of hysterectomy, who were randomized to CEE 0.625 mg/day (*n*  = 5310) or placebo (*n*  =5329). MHT increased the risk of stroke (HR 1.39, 95% CI 1.10–1.77) and VTE (1.33, 95% CI 0.99–1.7), without any effect on the risk of CHD and colorectal cancer. Interestingly, it decreased the risk of breast cancer (HR 0.77, 95% CI 0.59–1.01), hip (HR 0.61, 95% CI 0.41–0.91) and total fractures (HR 0.70, 95% CI 0.63–0.79) (44).

Nonetheless, an in-depth look into the WHI trials can reverse their first negative impression. One should take into consideration that the mean participants’ age was 63 years, with two-thirds being older than 60 years. When women were stratified according to their age, a marginally non-significant reduction in CHD risk was observed in the age group of 50–59 years (HR 0.56, 95% CI 0.3–1.03), with CEE alone, compared with no effect in the other age groups (60–69 and 70–79 years) (44). The respective absolute risk stratified by age was ten and five fewer cases for fatal and non-fatal CHD per 10,000 women/year for the ages of 50–59 and 60–69 years, respectively, in excess of four cases for those 70–79 years old (44). However, in the estrogen-alone arm, there was a significant reduction in a composite CHD outcome in those initiating treatment below age 60 years, and with long-term follow-up post-intervention there was a significant reduction in CHD events compared with placebo (43).

Moreover, a prospective Danish cohort (*n*  = 698,098 postmenopausal women, aged 51–69 years), published 5 years after the WHI, showed no increased risk of myocardial infarction (MI) (RR 1.03, 95% CI 0.95–1.11) for current MHT users compared with never users (45). In subgroup analyses, this risk increased with a longer duration (>4 years) (RR 1.81, 95% CI 1.19–2.77) (45). Interestingly, the risk for MI decreased (RR 0.62, 95% CI 0.42–0.93) with transdermal unopposed estrogen (compared with women who never used MHT) and it was also lower than that of oral regimen (45).

A Cochrane meta-analysis, published in 2015, showed a decreased risk of CHD (RR 0.52, 95% CI 0.29–0.96) and all-cause mortality (RR 0.70, 95% CI 0.52–0.95) if MHT was commenced within 10 years since menopause, raising the issue of ‘window of opportunity’ (46). No effect on the risk of stroke was observed, although the risk of VTE remained high (RR 1.74, 95% CI 1.11–2.73) (46).

The timing hypothesis was confirmed in more recent RCTs, such as the Early vs Late Intervention Trial with Estradiol study, in which 643 apparently healthy postmenopausal women were randomized to 17β-E_2_ (1 mg/day plus vaginal gel of progesterone for non-hysterectomized women) or placebo. After a median of 5 years, 17β-E_2_ decreased the rate of carotid intima-media thickness progression only in early postmenopausal women (<6 years since their FMP) compared with placebo. No difference in late postmenopausal women was observed in this regard (47). This cardioprotective effect of MHT in early postmenopausal women was replicated in the Danish Osteoporosis Prevention Study, including 502 women, 45–58 years old. According to its findings, 17β-E_2_ at a dose of 2 mg/day was associated with a 52% reduction in the risk of the composite CVD outcome (HR 0.48, 95% CI 0.26–0.87). There was no difference in the risk of VTE, stroke or breast cancer between groups (48).

However, another RCT, the Kronos Early Oestrogen Prevention Study, which recruited 728 women (42–58 years of age), failed to demonstrate any benefit of estrogen (either CEE 0.45 mg/day or transdermal 17β-E_2_ 50 μg/day) over placebo on coronary artery calcium score, another surrogate marker of atherosclerotic CVD. The duration of the trial was quite short (48 months) (49). Moreover, MHT has no effect on CVD risk in the setting of secondary prevention, as shown in the Heart and Estrogen/progestin Replacement Study (HERS) (50).

Based on the evidence presented above, most international societies converge regarding the indications for MHT (38, 51, 52). These include cases of EM or POI, as well as postmenopausal women <60 years old or within 10 years since menopause and at low-to-moderate CVD risk, for menopausal symptom relief, since the benefits of MHT outweigh potential risks (38, 51, 52). MHT is currently contraindicated in women at high CVD risk or for the sole purpose of primary or secondary prevention of CHD (38, 51, 52). In cases of moderate risk of CVD, transdermal estradiol should be preferred as first-line treatment, either alone for women without a uterus or in combination with micronized progesterone or dydrogesterone, due to their neutral effect on CVD risk factors and coagulation parameters (38). This is also the case for women at high VTE risk (38).

Clinicians should also consider that CVD mortality increases after MHT discontinuation, concerning either IHD (standardized mortality ratio (SMR) 1.26, 95% CI 1.16–1.37) or stroke (SMR 1.63, 95% CI 1.47–1.79), during the first post-treatment year. However, this risk is dissipated thereafter (SMR 0.75, 95% CI 0.72–0.78 and 0.89, 95% CI 0.85–0.94). This risk is also higher in women <60 years, but not in older women who discontinue MHT (SMR 1.94, 95% CI 1.51–2.48) (53).

The main concern with MHT is breast cancer risk, which is mostly attributed to progestogen. It is relatively lower with newer regimens, such as micronized progesterone and dydrogesterone (54) and seems to disappear after MHT discontinuation (38, 51, 52). According to a recent systematic review, MHT containing micronized progesterone does not increase breast cancer risk for up to 5 years of treatment. Limited evidence indicates an increased risk only if MHT is applied for >5 years (55). The key points regarding the effect of MHT on CVD risk are summarized in Table 1.

**Table 1:** The effect of MHT on CVD risk.

MHT improves lipid profile, glucose homeostasis and visceral adiposity. The evidence for an effect of MHT on BP and NALFD is inconclusive. Transdermal estradiol is preferred over oral regimens, since the former does not increase triglyceride concentrations and is not associated with increased VTE risk. MHT may reduce CVD morbidity and mortality, if commenced during the early postmenopausal period (<60 years or within 10 years since the FMP). In women with POI, MHT should be administered at least until the average age of menopause (50–52 years). CVD risk increases after MHT discontinuation. MHT is not currently recommended in women at high CVD risk or with a history of VTE or for the sole purpose of CVD prevention. The risk of breast cancer is minimized with the use of micronized progesterone or dydrogesterone.

BP, blood pressure; CVD, cardiovascular disease; FMP, final menstrual period; MHT, menopausal hormone therapy; NAFLD, non-alcoholic fatty liver disease; POI, premature ovarian insufficiency; VTE, venous thromboembolism.

## Special issues

Specific consideration should be paid to women with T2DM or dyslipidemia. In general, oral estrogens may be administered in peri-or recently postmenopausal women with new-onset T2DM and at low CVD risk. However, in the sub-population of obese postmenopausal women with T2DM and at moderate CVD risk, transdermal 17β-E_2_ is the preferred treatment, either as monotherapy or with a progestogen with minimal effects on glucose metabolism, such as micronized progesterone, dydrogesterone or transdermal norethisterone (56).

With respect to dyslipidemia, oral estrogens induce a more prominent effect on TC, LDL-C, Lp(a) and HDL-C concentrations, compared with transdermal ones. However, the latter should be used in women with hypertriglyceridemia (39). In any case, the 10-year risk of fatal CVD should be assessed to set the optimal LDL-C target and prescribe a lipid-lowering medication (i.e. statins, ezetimibe) when necessary (39). Regarding the progestogen, priority should be given to micronized progesterone or dydrogesterone, due to their neutral effect on lipid profile (39).

## Assessment of CVD risk in postmenopausal women

In general, clinicians need to consider the patient’s total CVD risk before initiating MHT. First, lipid profile (TC, LDL-C, TG and HDL-C), fasting plasma glucose and BP should be assessed in every postmenopausal woman. The next step is to estimate the woman’s 10-year risk for fatal CVD, according to the Systematic Coronary Risk Estimation (SCORE) system, proposed by the 2019 European Society of Cardiology / European Atherosclerosis Society guidelines (57). However, the SCORE system has some limitations, such as the sole inclusion of fatal ASCVD outcomes and the substantial variations of CVD risk across countries, which result in an underestimation of the individual’s risk. Therefore, it has recently been updated to SCORE2, for individuals aged 40–69 years (58), and to SCORE2-Older Persons (SCORE2-OP) risk model, for those >65 years old (59). This attempt was made on the basis of 10-year fatal and non-fatal ASCVD risk estimation in different European regions (58). Of note, non-HDL-C instead of TC is used in these two models (58, 59).

This updated SCORE is now recommended for CVD risk estimation in apparently healthy individuals without established ASCVD, DM, CKD, genetic lipid (FH) or BP disorders. These five states assign the patient at ‘very high’ or ‘high’ CVD risk (60). The former is considered in cases with established ASCVD, <50 years old with SCORE2 >7.5%, 50–69 years old with SCORE2 >10% or ≥70 years with SCORE2-OP >15% (60). On the other hand, an individual is considered at ‘high risk’ if his/her SCORE2 is 2.5–7.5%, 5–10% and 7.5–15% in cases of <50, 50–69 and >70 years of age, respectively (60). In ‘very high’ and ‘high’ risk patients, the LDL-C target is set at <55 mg/dL (1.4 mmol/L) and <70 mg/dL (1.8 mmol/L), respectively, with an additional need of ≥50% reduction in LDL-C concentrations (57). Furthermore, the term ‘low-to-moderate risk’ is used for women or men <50 years, 50–69 or ≥70 years with a SCORE2 or SCORE-OP of <2.5, <5 or <7.5%, respectively. Patients with well-controlled DM of <10 years duration, with no evidence of target organ damage (TOD) and no additional ASCVD risk factors, are classified as ‘moderate risk’ individuals (60).

Another widely used CVD risk calculator is the one proposed by the 2019 American College of Cardiology / American Heart Association guidelines (61). Notably, these consider POI as a CVD risk enhancing factor, which necessitates statin therapy in adults 40–75 years without DM and 10-year CVD risk of 7.5–19.9% (61).

Lp(a) assessment should also be assessed at least once in a person’s lifetime (57), since it may further increase ASCVD risk, in cases with concentrations >50 mg/dL (>120 nmol/L). Moreover, if these exceed 180 mg/dL (>430 nmol/L), the CVD risk is equivalent to that FH is associated with (57).

Statins, either alone or with ezetimibe, constitute the lipid-lowering treatment of choice in patients at ‘very high’ or ‘high’ ASCVD risk. In cases who cannot achieve these LDL-C targets, proprotein convertase subtilisin/kexin type 9 (PCSK-9) inhibitors (evolocumab or alirocumab) may be added. In cases with hypertriglyceridemia (135–499 mg/dL (1.5–5.6 mmol/L)), despite statin treatment, and high or very high CVD risk, a fibrate or high dose icosapentanyl-fatty acid (4 g/day) should be added (57). Statins may be also considered for individuals <40 years of age with DM and TOD or LDL-C >100 mg/dL (2.5 mmol/L) (60).

In all cases, adoption of a Mediterranean dietary pattern, regular exercise, smoking cessation and alcohol restriction to a maximum of 100 g/week, is recommended (60). BP should be lowered to <140/90 mm Hg in all patients. In treated patients aged <69 years, the target range of systolic BP (SBP) is 120–130 mm Hg, whereas in those ≥70 years, the goal for SBP is <140 mm Hg or even <130 mmHg, if tolerated. Diastolic BP should be lowered to <80 mm Hg in all treated patients (60). In patients with DM at ‘high’ or ‘very high’ CVD risk, low-dose aspirin may be administered for primary prevention (60).

**Figure 1:**
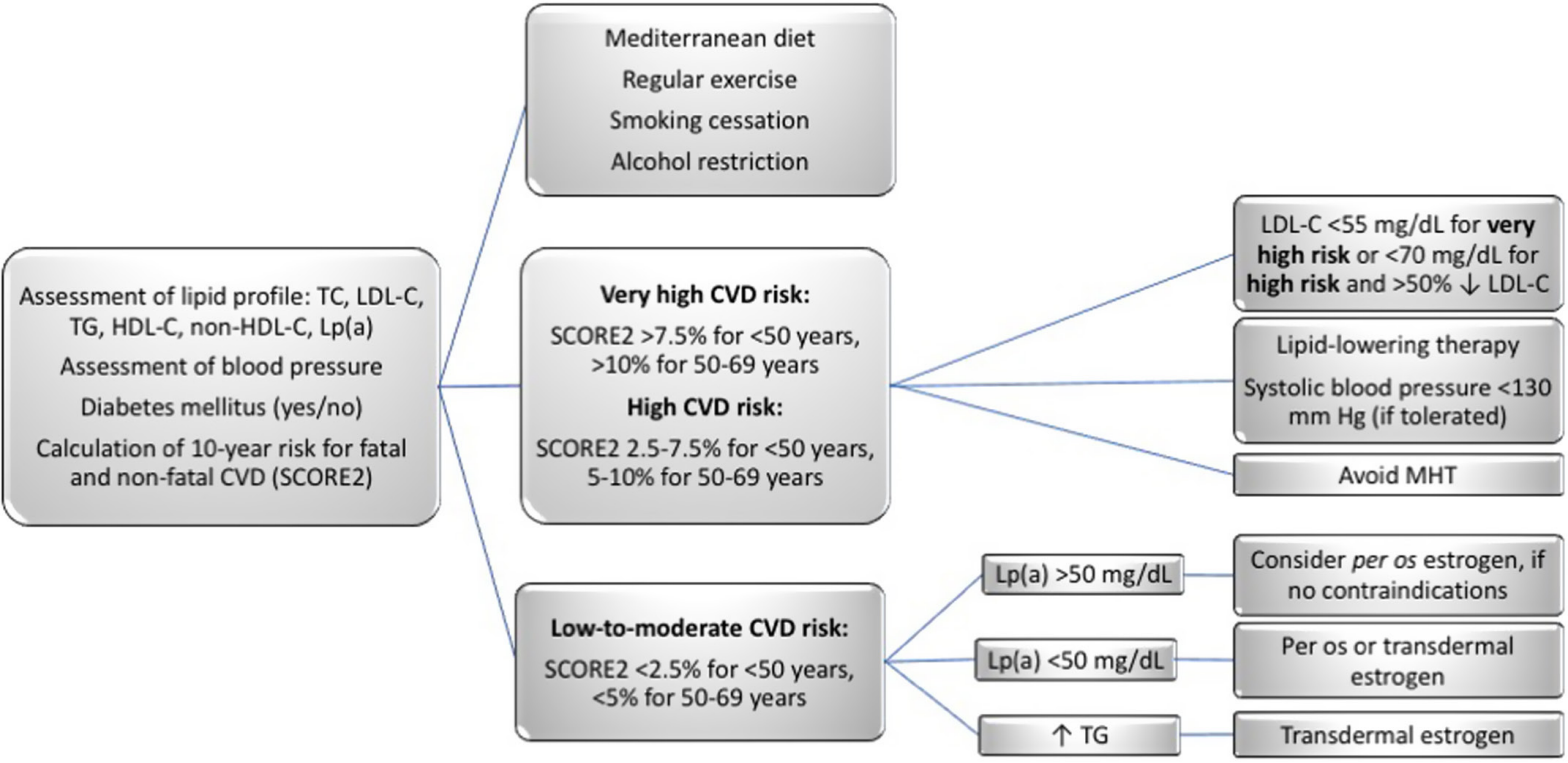
Algorithm of CVD risk assessment and personalized intervention in postmenopausal women. Estrogen – based treatment is indicated for women with bothersome menopausal symptoms within 10 years of their final menstrual period or to women with premature ovarian insufficiency or early menopause. CVD, cardiovascular disease; HDL-C, high-density lipoprotein cholesterol; LDL-C, low-density lipoprotein cholesterol; Lp(a), lipoprotein(a); MHT, menopausal hormone therapy; SCORE, Systematic Coronary Risk Estimation; TC, total cholesterol; TG, triglycerides.

Based on these guidelines, an algorithm of CVD risk assessment and personalized intervention in postmenopausal women aged <50 or 50–69 years old, is illustrated in Fig. 1.

## Conclusions

In conclusion, transition to menopause predisposes the woman to increased CVD risk, due to visceral obesity, atherogenic dyslipidemia, dysregulation in glucose homeostasis, NAFLD and hypertension. However, whether menopause *per se* is associated with a higher risk of CVD events has not been proven. On the other hand, both EM and POI are associated with increased CVD morbidity and mortality, mainly attributed to IHD. MHT ameliorates most of the traditional CVD risk factors, with different effects, depending on the type, dose, route of administration and type of progestogen. MHT may reduce the risk of CVD events if prescribed within 10 years since the FMP or in postmenopausal women <60 years old and at low-moderate CVD risk. However, MHT should currently not be prescribed for the sole purpose of CVD prevention. In any case, there is an exigent need for well-designed RCTs with the newer regimens, such as transdermal estrogen and micronized progesterone, to prove their efficacy and safety in terms of CVD and breast cancer risk.

## Declaration of interest

Prof. Stevenson has received grants/research support from Abbott, Mylan and Pfizer; consulting fees from Abbott, Mylan and Pfizer; and speaker’s honoraria from Abbott, Bayer, Gedeon Richter, Menarini, Mylan, and Pfizer. The other authors declare that there is no conflict of interest that could be perceived as prejudicing the impartiality of the research reported.

## Funding

This work did not receive any specific grant from any funding agency in the public, commercial or not-for-profit sector.
